# Men’s understanding of three different measures of transactional sex: A cognitive interviewing study among men in Rakai, Uganda

**DOI:** 10.21203/rs.3.rs-6465876/v1

**Published:** 2025-05-02

**Authors:** Holly Nishimura, Neema Nakyanjo, Waru Gichane, William Ddaaki, Anne Kiyingi, Emmanuel Mukwana, Fred Nalugoda, Charles Ssekyewa, Julie Denison, M. Kate Grabowski, Joseph Kagaayi, Caitlin E. Kennedy

**Affiliations:** Department of International Health, Johns Hopkins Bloomberg School of Public Health, Baltimore, MD; Rakai Health Sciences Program, Kalisizo, Uganda; Department of Obstetrics, Gynecology and Reproductive Sciences, University of California San Francisco, San Francisco, CA, USA; Rakai Health Sciences Program, Kalisizo, Uganda; Rakai Health Sciences Program, Kalisizo, Uganda; Rakai Health Sciences Program, Kalisizo, Uganda; Rakai Health Sciences Program, Kalisizo, Uganda; Rakai Health Sciences Program, Kalisizo, Uganda; Department of International Health, Johns Hopkins Bloomberg School of Public Health, Baltimore, MD; Department of Pathology, Johns Hopkins University School of Medicine, Baltimore, MD, USA; Rakai Health Sciences Program, Kalisizo, Uganda; Department of International Health, Johns Hopkins Bloomberg School of Public Health, Baltimore, MD

**Keywords:** transactional sex, men, Uganda, cognitive interviews, measurement

## Abstract

Lack of valid and reliable measures and inconsistent transactional sex (TS) measurement has resulted in poor understanding of the practice, particularly among men. To improve TS measurement and facilitate comparison across studies, we examined how men comprehend and respond to three common TS measures.

We conducted cognitive interviews with 25 sexually active adult men in Rakai, Uganda from November 2021-February 2022 and thematically analysed the data.

Most men responded affirmatively to the three TS measures, but there was variation in responses across measures. The two measures that assessed relationship motivation and gendered norms of material provision for sex showed better comprehension and consistency than the measure without these components. For these two measures, a substantial proportion of men responded affirmatively but provided explanations related to gendered expectations of material provision rather than describing provision in a specific relationship which was not the intent of the measures. The measure developed by Wamoyi and colleagues appeared to most accurately measure TS.

Our findings support TS measures that include a clear statement of motivation and account for gendered norms of giving and receiving. Given heterogeneity in TS measurement, this study enhances our understanding of common TS measures.

## Introduction

Transactional sex relationships are non-commercial, non-marital relationships motivated by the implicit exchange of goods, money, or favours for sex (Stoebenau et al. 2016). Research and interventions addressing transactional sex have increased in recent years because transactional sex is thought to be associated with unprotected sex, and unequal relationship power dynamics which contribute to increased risk of HIV among adolescent girls and young women (AGYW) in sub-Saharan Africa (Stoebenau et al. 2016). As research on transactional sex has proliferated, so have inconsistencies in measurement, limiting our understanding of the practice and its relationship to HIV (Wamoyi et al. 2019). Wamoyi et al. (2019) identified several flaws with transactional sex measurement which could compromise validity of findings. First, transactional sex measures often conflate transactional sex with commercial sex work and do not differentiate between marital and non-marital relationships. Second, transactional sex measures have failed to account for gendered norms of giving and receiving or relationship motivations which has resulted in misclassification of transactional sex relationships. Identification of transactional sex relationships through valid and reliable measurement is critical to understand the extent to which transactional sex impacts risk for HIV and to evaluate the impact of interventions addressing transactional sex.

Most research on transactional sex has focused on AGYW; there has been substantially less research on men and boys engaged in transactional sex. Estimates of transactional sex prevalence among men and boys vary widely across settings, and the relationship between HIV transmission and transactional sex among men is inconclusive (Wamoyi et al. 2016). In addition, transactional sex measures often fail to assess whether men are providers of money or material items in the relationship, which may indicate unequal relationship power (Wamoyi et al. 2016). Heterogeneity in transactional sex measurement may account for some of the variation in prevalence estimates and the lack of consistent measurement has made comparisons across studies challenging (Wamoyi et al. 2016).

Wamoyi et al. (2019) developed improved transactional sex measures and validated them in a study in Tanzania and Uganda. Their goal was to more accurately identify transactional sex relationships and provide recommendations to guide future development of valid and reliable transactional sex measures. These recommendations were: 1) to clearly differentiate transactional sex from sex work; 2) exclude marital relationships 3) include a clear statement of the motivation for the sexual relationship; 4) account for gendered expectations of giving and receiving in relationships; and 5) ensure wording is non-judgmental. We sought to further this work by using cognitive interviewing to compare responses to and understanding of the measure developed by Wamoyi et al. (2019) to two commonly used transactional sex measures in a diverse sample of adult men residing in Rakai, Uganda.

## Methods

### Study Setting

Rakai District is located in south-central Uganda. The district is bordered to the south by Tanzania, to the north by Masaka District, and to the east by Lake Victoria. The region is mostly rural and characterized by three main community types: agricultural, trading centers, and fishing villages. Adult HIV prevalence ranges from 9% in agrarian communities to 43% in fishing communities (Chang et al. 2016).

### Sampling and Recruitment

We conducted a qualitative study among sexually active adult men ages 18–49, residing in Rakai District, Uganda enrolled in the Rakai Community Cohort Study (RCCS). RCCS is an open population-based cohort which enrols all consenting adult residents aged 15–49 years in approximately 50 communities distributed throughout Rakai District. Interviews were conducted between November 2021 and February 2022. Prior to recruitment, we conducted three pilot interviews to refine translation of transactional sex items to Luganda. We recruited a total of 27 men who were purposively sampled based on their responses to measure 1 (Table 2) in the RCCS survey in 2018. Two recruited men declined to participate. We selected 20 men who responded “yes, gave only” to measure 1 and five men who provided any other response. Participants were sampled for variation based on geographical location across the Rakai region (fishing, trading, and agrarian communities), age, education level, and occupation. Men residing in agrarian and trading communities were contacted via mobile phone to assess their interest in participating in a study to understand non-marital sexual partnerships. Men residing in fishing villages, who were more challenging to recruit because they are highly mobile and spend long periods of time fishing without access to phones, were mobilized by RCCS staff who provided a mobile phone and private location for recruitment conversations and interviews.

### Measure Selection

Measures 1 and 2 were selected because they have been used in several rounds of RCCS and measures with the exact or similar wording have been used in a wide range of prior transactional sex studies (Gavin et al. 2006; Rositch et al. 2012; Serwadda et al. 1992; Shaffer et al. 2010; Hunter et al. 1994; Dunkle et al. 2004; Mmbaga et al. 2007; Kwena et al. 2012; Pettifor et al. 2005; Ranganathan et al. 2017; Jewkes et al. 2006a, b).

Measure 1 was added to the RCCS in early rounds of the survey and intended to identify relationships in which material exchange occurred. A single item is used for both men and women. Initially response options were “yes” or “no” but were later changed to specify direction of giving and receiving (yes, gave and received; yes, gave only; yes, received only; no). Measure 2 is also a single item measure but is phrased differently for men and women. Measure 2 was developed by RCCS researchers and was added to the questionnaire in 2015 (Round 17) to assess transactional sex based on publications presenting refined definitions of the term. In 2016, Stoebenau and colleagues published a conceptual paper arguing for the adoption of this, now widely used, transactional sex definition: “non-commercial, non-marital sexual relationships motivated by the implicit assumption that sex will be exchanged for material support or other benefit” (Stoebenau et al. 2016). Administration of measure 2 in RCCS interviews was conditional on an affirmative response on measure 1. This measure was removed from the RCCS in 2021 (Round 20). An improved single-item transactional sex measure was developed by Wamoyi et al. (2019) which referenced gendered expectations for giving and receiving, explicitly excluded relationships with sex workers, and asked about relationship motivations. This measure is considered the current “gold standard” for transactional sex measurement and was added to the RCCS in November 2021.

### Cognitive Interviews

Cognitive interviewing is a qualitative method applied to the development and adaptation of questionnaires and other self-report measures (Willis 2004). Cognitive interviews empirically study how individuals mentally process and respond to survey measures. The interviews rely on intensive verbal probing of participants by a trained interviewer. Because our aim was to understand the meaning of existing transactional sex measures common in the extant literature, we only conducted one round of interviews and deviated from other cognitive interviewing approaches which revise and retest measures (Willis 2004).

Two Rakai Health Sciences Program (RHSP) staff with training in qualitative data collection and cognitive interviewing techniques conducted interviews via mobile phone. During the interviews, each participant was asked to recall their last non-marital, non-commercial sexual relationship in the past 12 months and then respond to three different transactional sex measures (Table 1) based on this specific relationship. We removed measures 1 and 2 for the last four (IDI 26–29) participants to gain insight into whether answering transactional sex measures 1 and 2 influenced responses to measure 3 and looked for consistencies and inconsistencies across interviews. The interviewers were given scripted verbal probes and were trained to incorporate spontaneous verbal probes when appropriate. Scripted verbal probes included:

- Please repeat the question I just asked in your own words.
- Tell me about how you arrived at your response.
- How easy or difficult was this question to answer?

### Data Analysis

A deductive and inductive thematic analysis approach was used based on the interview guide and close review of the transcripts. We looked for patterns in respondents’ understanding of transactional sex measures and response patterns by age and education. Participant quotes were categorized into themes and were used to understand participant comprehension of the measures, issues with the measures and response options, and fidelity with Wamoyi et al.’s recommendations for transactional sex measure development (Wamoyi et al. 2019). These recommendations were: 1) to clearly differentiate transactional sex from sex work; 2) exclude marital relationships 3) include a clear statement of the motivation for the sexual relationship; 4) account for gendered expectations of giving and receiving in relationships; and 5) ensure wording is non-judgmental. We also attempted to identify patterns in participant explanations with discordant responses to the three measures.

### Ethical Considerations

This study was approved by the Uganda Virus Research Institute Research and Ethics Committee, the Uganda National Council for Science and Technology, and Johns Hopkins Bloomberg School of Public Health Institutional Review Board.

## Results

Table 2 shows the demographic characteristics of study participants and their responses to each of the three measures. Below, we present the patterns in response explanations by measure and response option. We also summarize issues participants encountered in responding to each measure. Most participants responded “Yes” to all 3 items. Three participants had discordant responses across the three items (IDI 07, IDI 15, IDI 16); we present an analysis for these three individuals in Table 3.

### Measure 1 (RCCS): Were money, gifts, or favours ever exchanged for sex with this partner?

Of the 21 respondents asked measure 1, approximately half (n = 10) responded “Yes, gave only.” These responses were categorized as transactional sex. Eight men responded “yes, gave and received,” two responded “no,” a response for one was not recorded and none responded “yes, received only.”

### Measure 1 Response Explanations

Regardless of participants’ responses to measure 1, the measure was often not understood as intended. We describe below common themes in men’s understanding of measure 1 and explanation for their responses.

#### Measure 1 response: Yes, gave only.

Among participants who responded “Yes, gave only,” half described a non-commercial, non-marital sexual relationship in which material exchange for sex was a primary motivation. In the example, below a man describes material provision as the reason for starting a relationship with his sexual partner. For these participants, the measure correctly classified relationships as transactional sex.

I decided to start enticing her by buying her things that I saw she needed. There’s one day I bought her shoes, and I took them to her. I continued to buy her many things like clothes, so she realized that she was no longer using her salary to buy the basic things that she wanted. And I was giving her everything. So, she started to get used to me and asked me why I cared about her so much and if I will continue to buy for her the things that she wants. I told her I will and that’s how we started to have a sexual relationship. IDI 19

The other half of participants who responded “Yes, gave only” described provision in terms of gendered norms of giving and receiving in sexual relationships. For example, one respondent said, “Women say that we are the ones supposed to provide. Even if the woman has more, she expects the man to be the one to give to her.” According to these respondents, provision of money by the male partner was expected by women, the community, and men themselves. It is unclear whether men who responded to measure 1 in terms of gender norms were referencing their transactional sex relationship; therefore, there is a risk of misclassification of non-transactional sex relationships as transactional sex. For example,

Participant: Because I loved her, I gave her a gift and she also gave me what I wanted.

Interviewer: What do you mean when you say she gave you what you wanted?

Participant: I was able to have sex with her. I gave her my gift and she also gave me hers. IDI 18

#### Measure 1 response: Yes, gave and received.

Participants’ explanations for responding “Yes, gave and received” fell into two major categories. First, some participants misinterpreted the measure as asking about mutual exchange of material items. For example, this quote illustrates how the participant interpreted the question as whether there was mutual exchange of gifts between himself and his partner rather than exchange of these items for sex: “That exchange occurred during Valentine’s Day. I gave her a gift during Valentine’s Day and she gave me a gift on my birthday.” In this category, it is unclear whether the relationship described would be considered transactional sex. In the second category, participants interpreted the measure as asking whether the man gives material items and, in turn, receives sex. Based on this explanation, “Yes, gave only” would have been the expected response for measure 1 rather than “Yes, gave and received”. In this category, there is potential misclassification of transactional sex relationships as non-transactional sex.

#### Measure 1 response: No.

Two participants responded “No” to measure 1. One respondent said that there was no exchange in either direction in the relationship, suggesting the measure correctly identified this relationship as non-transactional sex. The other respondent who answered “No” explained that he provided material items to show he cares for his partner and was not a direct exchange for sex. This respondent could have interpreted measure 1 as asking about an exchange that occurred at the same time as sex or could have been describing a non-materially motivated relationship. I have never done it as a way of paying her to have sex, but as you’re in a relationship as people who love each other you may give your partner some money if she tells you that she needs to buy something. But not paying her to have sex at that time. IDI 20

#### Issues with measure 1 and response options.

Despite frequent misinterpretation of measure 1 as intended, most participants said the measure was easy to answer. Explanations for the ease of answering the question were related to being able to recall a specific event in which an exchange occurred and trusting in the confidentiality of the interview.

It was not difficult at all because it was the truth, and I am sure of it. It happened and I am the one who did it, so I know it very well and I know what I am talking about. IDI 18

Two respondents described difficulties responding to measure 1 related to recalling a past sexual relationship and too many response options to choose from. For example, one man said, “I don’t have an official sexual partner right now, that is where I am finding some challenge.”

### Measure 2: Is the primary reason you had a sexual relationship with this partner because you provided material support to her (such as money for personal needs, looking after your children, paying your rent, starting a business etc.)?

Compared to measure 1, measure 2 generated more responses that would be classified as transactional sex. Sixteen participants responded “Yes”. These responses were categorized as transactional sex. Five men responded “No”.

### Measure 2 response explanations

Most participants understood measure 2 as intended regardless of whether they responded “Yes” or “No” to the question. Participants indicated that they were able to identify the “primary reason” for having a sexual relationship with their partner. Participants who responded “Yes” were able to recall and describe characteristics of the relationship which indicated that the motivation for the relationship was provision of material support to their partner.

#### Measure 2 response: Yes.

Some participants who reported “Yes” to measure 2 clearly described sexual relationships motivated by material exchange.

You see she told me that if I don’t help her with her problems, we shall not have a sexual relationship because she needed a phone, and she wanted to have a smart phone just like her friends. So, she told me if I wanted to have a sexual relationship with her, I must buy her a smart phone. IDI 24

As with measure 1, some participants who responded “Yes” responded generally based on gendered norms of male provision. It was not clear if the response would have been “Yes” for the specific relationship they were asked to recall, and these responses may have misclassified non-transactional sex relationships as transactional sex.

You can’t pursue a woman with nothing. It’s not proper - if you are pursuing a woman you need to give her something that entices her. You see you can pursue a lady who is wealthier or has more money but because of love, you will still buy her something that entices her. IDI 05

A small number of participants described giving material support to their partner as a way to show they care but did not connect provision as a primary motivation for the sexual relationship in their explanation. It is not clear, based on participant explanations, whether these relationships would be considered transactional sex.

#### Measure 2 response: No.

Descriptions of relationships from participants who responded “No” to measure 2 indicated that material exchange was not the primary motivation for the relationships and were consistent with descriptions of non-transactional sex relationships. For example,

… our sexual relationship was not based on either gifts or money, we were just friends and we ended up being in a sexual relationship, but it’s based on mutual understanding not on gifts or money.

IDI 15

One respondent initially responded “No” but upon further probing on relationship motivations, changed his response to “Yes,” suggesting that there could be some misclassification of transactional sex relationships as non-transactional sex associated with measure 2.

#### Issues with measure 2 and response options.

Most participants said measure 2 was easy to answer. Similar to measure 1, explanations for the ease of answering the question were related to being able to recall a specific event or relationship in which an exchange occurred. Three respondents described difficulties responding to measure 2 related to recalling a past sexual relationship, the measure being too long, or interpreting the item as three different questions.

### Measure 3: Have you given a partner any money, gifts, or helped/supported her to pay for things mainly to start or continue a sexual relationship with her?

Twenty participants responded “Yes” and their relationships were categorized as transactional sex measure 3. Four participants responded “No”.

### Measure 3 response explanations

Most participants appeared to understand measure 3 as intended regardless of whether they responded “Yes” or “No.” Participants’ explanations indicated that they understood motivations for starting or continuing the relationship and could discern whether motivations were related to material support. The four participants who were only asked measure 3 also appeared to interpret the question as intended, suggesting that responses to measure 3 were unlikely to be influenced by answering measures 1 and 2.

#### Measure 3 response: Yes.

Most participants who reported “Yes” to measure 3 clearly described sexual relationships motivated by material exchange.

…I first gave her various gifts and I used to take her to hangout places. I used to give her those gifts with the aim of starting a sexual relationship with her. I first became her friend then started to pursue her with gifts, so she later accepted, and we became boyfriend and girlfriend.

IDI 26

Only one participant who responded “Yes” described motivations for provision that did not align with transactional sex. This could indicate potential misclassification of non-transactional relationships as transactional sex associated with this measure. Another category of relationships for which men responded “Yes” involved provision as a way of “enticing” partners. It is not clear whether this practice is indicative of transactional sex or normative courtship behaviour for men.

I have done it; I have given her something. As you know, a person you have never known before, you need at least to give in something to her as a way of trying to find a way of getting closer to her mind. IDI 10

#### Measure 3 response: No.

All participants who responded “No” to measure 3 described sexual relationships that would not be defined as transactional sex.

You see you first ask the person to become your partner. If she accepts and stays with you for some time, you give her something not because you’re having sex with her but because you’re giving to her as a friend. If you have spent time with your partner, then you can give her money and gifts to keep her happy. The truth is you must give her some money but not because you want to start a sexual relationship, but as a way of appreciating her. IDI 16

#### Issues with measure 3 and response options.

Most participants said measure 3 was easy to answer. Explanations for the ease of answering the question were related to being able to recall a specific event or relationship in which an exchange occurred. Two respondents described difficulties responding to measure 3 related to discomfort discussing sexual relationships with a stranger and the problems with the length of measure 3.

### Comparison of three transactional sex measures

**Figure 1.**
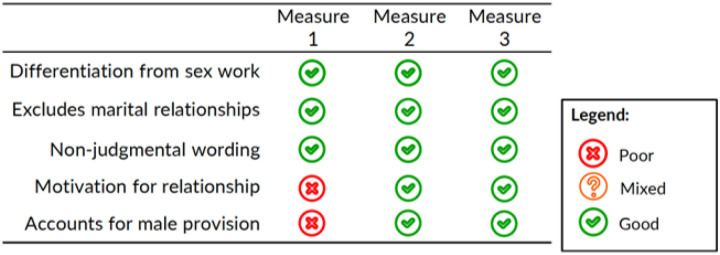
Assessment of three transactional sex measures

Figure 1 summarizes the performance of the three transactional sex measures in terms of the five recommendations for transactional sex measurement set forth by (Wamoyi et al. 2019) and other performance considerations.

#### Alignment with Wamoyi et al.’s 5 recommendations for transactional sex measurement.

Wamoyi et al. (2019) propose five recommendations for transactional sex measurement: 1) to clearly differentiate transactional sex from sex work; 2) exclude marital relationships 3) include a clear statement of the motivation for the sexual relationship; 4) account for gendered expectations of giving and receiving in relationships; and 5) ensure wording is non-judgmental. Below, we discuss alignment of the three transactional sex measures with these five recommendations.

#### Alignment with recommendations 1 & 2.

Measures 1, 2, and 3 do not differentiate between sex work or marital relationships on their own. To identify non-commercial, non-marital relationships, we asked participants to recall a sexual relationship in the past 12 months with a partner that was not their wife and not a sex worker. This approach appeared to be effective as none of the participants described marital or commercial relationships.

#### Alignment with recommendation 3.

Measure 1 did not include any reference to relationship motivation. Measures 2 and 3 included clauses that described the motivation for the relationship which appeared to improve measure comprehension.

#### Alignment with recommendation 4.

Measures 2 and 3 also accounted for gendered norms of provision specific to men by explicitly asking if the man gave material/financial goods to the woman, while measure 1 referred to the direction of giving and receiving but was not gender-specific. Inclusion of a gender norms clause also contributed to improved construct measure comprehension.

#### Alignment with recommendation 5.

We pilot tested measures with three participants to ensure measures were translated correctly and were culturally appropriate. Though not asked directly, participants did not describe feeling that the phrasing of the measures was judgmental. More than the wording of the question, however, rapport with the interviewer and trust that confidentiality would not be breached was important in obtaining responses from participants.

### Other performance considerations

**Figure 2.**
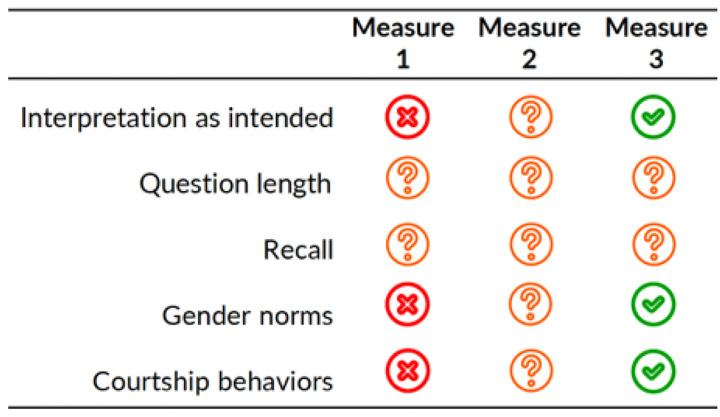
Other performance considerations for three transactional sex measures

We assessed consistency of interpretation across participants and measures, interpretation of measures as intended, measure length, and whether the measure appeared to distinguish courtship norms from transactional sex. Results are illustrated in Fig. 2.

#### Consistent interpretation across participants and measures.

Participant responses were highly variable in the interpretation of measure 1 which was more susceptible to being misinterpreted than measures 2 and 3. In our analysis of discordant responses, we found that all three respondents with discordant responses reported “Yes” to measure 1 and “No” to measures 2 and 3. Responses also indicated that measures 2 and 3 had more similar interpretations. For all respondents, responses were consistent across measures 2 and 3. If all three measures reliably measured the same construct, transactional sex, we would expect answers to be consistent across the three items.

#### Interpretation of measures as intended.

At face value, measures 2 and 3 appear to measure different constructs. Measure 2 asks men to report whether obtaining sex was the primary motivation of providing materials to their partners while measure 3’s focused on participant’s actual provision and motivations. Despite this difference, measures 2 and 3 showed similar performance in differentiating between transactional sex and non-transactional sex relationships. Though rare, measure 2 also appears to reflect men’s beliefs about provision in relationships.

#### Measure length.

Across all items, participants had trouble with recall if they were not currently in a relationship and there were complaints that all three measures were either too long and that measure 1 had too many response options.

#### Differentiation of courtship norms from transactional sex.

None of the measures in this study distinguish between transactional sex and courtship norms. A notable proportion of participants who responded “Yes” to measures 2 and 3 described sexual relationships in which giving at the start of a relationship appeared to be normative courtship behaviour and a demonstration of men’s ability to be a good provider.

## Discussion

We analysed how a sample of men in Uganda understood and responded to three transactional sex measures. We identified significant issues with the interpretation of measure 1 but measures 2 and 3 were generally interpreted as intended by our participants. Measure 1 may identify some transactional sex relationships but may be a better indicator of men’s beliefs about gendered expectations of giving and receiving in romantic relationships. The measure appears to have poor specificity in identifying transactional sex relationships and may overestimate transactional sex compared to measures 2 and 3. Measure 3, which was developed by Wamoyi et al. (2019), performed best in terms of consistent interpretation across measures and measure comprehension. Our findings support recommendations for transactional sex measures to specify the primary motivation for the relationship and account for gendered norms of giving and receiving in sexual relationships.

We noted that participants had difficulties distinguishing between transactional sex and courtship norms with all three measures. A large body of literature on transactional sex in Africa, has highlighted the inextricable linkages between sex, love, and material provision in intimate relationships (Stoebenau et al., 2016). While adding the phrase, “mainly in order to” in Wamoyi et al. (2019) transactional sex measure was intended to make this distinction, we found that participants’ responded to some measures based on how men are generally expected to provide to initiate a relationship or attract a partner, rather than material provision in a specific relationship. To focus participants on provision as a means for having a sexual relationship, we recommend phrasing measures so that the relationship motivation clause precedes the provision clause. For example, the measure developed by Wamoyi and colleagues (2019) could be rephrased to, “Mainly in order to start or continue a sexual relationship with a partner (motivation clause), have you given her any money, gifts, or helped/supported her to pay for things (provision clause)?” Further refinement and testing of transactional sex measures, particularly across different languages, is needed to address this issue.

A strength of this research is the use of cognitive interviewing which has not been widely used in global health research. The method is particularly important for measurement of complex concepts, like transactional sex, which might not be well understood (Scott, Ummer, and LeFevre 2021) and is stigmatized in some contexts.. Cognitive interviewing can also be useful in global health research to refine translation of items for use in different settings. Findings from cognitive interviews can help avoid measurement error by ensuring that questions are comprehended by participants as intended and that respondents can answer accurately. By testing the transactional sex items with participants of various ages, geographic and educational backgrounds, we can provide further support of the transferability of Wamoyi et al.’s transactional sex measure. We also find value in cognitive interviewing for interpreting and contextualizing findings from measures already in use that may not have undergone prior cognitive testing. This may be useful for secondary analyses of earlier waves of RCCS data or other studies that use similar transactional sex items.

This study has limitations. The verbal probes employed in this study required participants to think and verbalize in a manner that was unfamiliar to many, and several participants had difficulty understanding the cognitive interviewing questions. Participants were told prior to hearing each item that they would be asked to repeat the item in their own words and therefore may been listening more carefully than in an actual interview setting. In addition, participants’ ability to repeat the question in their own words may not have been related to question comprehension. Social desirability bias likely influenced participants’ answers about ease of answering questions, as most participants said the questions were easy to answer.

## Conclusion

Our findings shed light on how men comprehend and respond to three common transactional sex measures. We found that the item developed by Wamoyi et al. (2019) performed best in terms of interpretation of the measure as intended and incorporation of recommendations for transactional sex measure development. Our findings strongly support recommendations to include a clear statement of motivation and account for gendered norms of giving and receiving in romantic and sexual relationships. Given the heterogeneity in transactional sex measurement, this study enhances our understanding of previously used transactional sex measures which may facilitate comparison across transactional sex studies among men. Further study should examine how to refine transactional sex measures to differentiate transactional sex from normative courtship behaviours and how to improve item comprehension in research settings.

## Figures and Tables

**Table 1:** Transactional sex measures in English and Luganda

Measure	English and Luganda translations
Measure 1 (RCCS)	Were money, gifts, or favors ever exchanged for sex with this partner? Yes, received only; Yes, gave only*; Yes, gave & received; No Omuntu ono wali omuwaddeyo oba yali akuwaddeyo ku sente oba ekirabo okwegatta naye? Yee, nz’eyafuna; Yee, Nz’eyagaba*; Yee, nagaba era nenfuna; Nedda
Measure 2 (RCCS)	Is the primary reason you had a sexual relationship with this partner because you provided material support to her (such as money for personal needs, looking after your children, paying your rent, starting a business etc.)? Yes*; No Ensonga enkulu eyakuleetera okwetaba mu mukwano ogulimu okwegatta n’omwagalwawo kyalilwakuba nti wamuyambako mubyetaago bye? okugeza nga okumuwa sente asobole okumala ebyetaago, okulabirila abaana be, okusasula sente z’obupangisa oba okutandika wo ka business? Yee*; Nedda
Measure 3 (Wamoyi et al., 2019)	Have you given a partner any money, gifts, or helped/supported her to pay for things mainly to start or continue a sexual relationship with her? Yes*; No Wali owadde muganziwo atali mukyalawo era nga sineko sente, ebirabo, oba okumuyambako okusasula ebintu nga ekigendererwa ekikulu kutandikawo enkolagana ey’omukwano ogulimu okwegatta oba okugenda mu maaso n’omukwano guno. Yee*; Nedda

Note: Relationships in which respondents provided responses with an (*) were classified as transactional sex relationships.

**Table 2.** Participant demographic characteristics and measure responses, N=25

IDI#	Age	Education	Marital status	Occupation	Community	Response: Measure 1	Response: Measure 2	Response: Measure 3
IDI04	35–44	Primary	Married	Other	Fishing	Yes, gave only	Yes	Yes
IDI05	25–34	Primary	Never married	Fishing	Fishing	Yes, gave only	Yes	Yes
IDI06	18–24	Primary	Never married	Fishing	Fishing	Yes, gave only	Yes	Yes
IDI07	35–44	Primary	Married	Military/police	Trading	Yes, gave only	No	No
IDI09	35–44	Primary	Married	Fishing	Fishing	Yes, gave & received	Yes	Yes
IDI10	25–34	Secondary	Married	Fishing	Fishing	Yes, gave only	Yes	Yes
IDI11	35–44	Primary	Never married	Fishing	Fishing	Yes, gave only	Yes	Yes
IDI12	25–34	Primary	Married	Fishing	Fishing	Yes, gave & received	Yes	Yes
IDI13	18–24	Primary	Never married	Student	Trading	Yes, gave & received	Yes	Yes
IDI14	25–34	Secondary	Married	Boda boda	Trading	Yes, gave only	Yes	Yes
IDI15	18–24	Secondary	Previously	Student	Agrarian	Yes, gave & received	No	No
IDI16	25–34	Secondary	Married	Trading	Agrarian	Yes, gave only	No	No
IDI17	18–24	Primary	Never married	Student	Trading	No	No	No
IDI18	≥45	Secondary	Married	Construction	Agrarian	Yes, gave & received	Yes	Yes
IDI19	18–24	Primary	Previously	Agriculture	Agrarian	N/R	Yes	NR
IDI20	18–24	Primary	Previously	Boda boda	Agrarian	No	No	No
IDI21	35–44	Secondary	Married	Fishing	Fishing	Yes, gave & received	Yes	Yes
IDI22	25–34	Secondary	Married	Boda boda	Fishing	Yes, gave & received	Yes	Yes
IDI23	18–24	Primary	Previously	Construction	Agrarian	Yes, gave & received	Yes	Yes
IDI24	18–24	Secondary	Previously	Agriculture	Trading	Yes, gave only	Yes	Yes
IDI25	≥45	Primary	Never married	Agriculture	Fishing	Yes, gave only	Yes	Yes
IDI26	35–44	Secondary	Married	Military	Trading	--	--	Yes
IDI27	35–44	Primary	Never married	Agriculture	Agrarian	--	--	Yes
IDI28	25–34	Primary	Married	Trucker	Trading	--	--	Yes
IDI29	25–34	Primary	Previously	Other	Agrarian	--	--	Yes

Notes: N/A: not applicable; N/R: no response; IDI26–29, respondents were only asked measure 3.

## Data Availability

The participants of this study did not give written consent for their data to be shared publicly, so due to the sensitive nature of the research supporting data is not available.
